# Correlation between uniocular deviation and duction changes following different decompression surgeries in thyroid eye disease

**DOI:** 10.1186/s12886-021-01892-9

**Published:** 2021-03-12

**Authors:** Jie Guo, Xiaofeng Li, Ruiqi Ma, Jiang Qian

**Affiliations:** 1 Eye Institute and Department of Ophthalmology, Eye & ENT Hospital, Fudan University, Shanghai, China; 2 NHC Key Laboratory of Myopia (Fudan University, Key Laboratory of Myopia, Chinese Academy of Medical Sciences, No. 83 Fenyang Road, Shanghai, 200031 China; 3 Laboratory of Myopia, Chinese Academy of Medical Sciences, No. 83 Fenyang Road, Shanghai, 200031 China

**Keywords:** Thyroid eye disease, Orbital decompression, Ocular duction, Strabismus

## Abstract

**Background:**

Postoperative ocular imbalance is an important problem for orbital decompression surgery in thyroid eye disease (TED). The aim of this study was to evaluate the changes in unilateral ocular deviation and duction following orbital decompression and discuss the biomechanics of ocular imbalance.

**Methods:**

Fifty-four TED patients who underwent unilateral orbital decompression were included. Fifteen patients underwent 1-wall (deep lateral wall) decompression, 18 patients underwent 2-wall (deep lateral and medial wall) decompression and 21 patients underwent 3-wall (deep lateral, medial and inferior wall) decompression. Objective and subjective deviation of the operated eyes were evaluated using the prism test and synoptophore, respectively. Ocular ductions were measured using Hirschberg’s method. The diameters of the extraocular rectus were measured by computed tomography.

**Results:**

Ocular deviation and duction showed no significant difference after 1-wall decompression (*p* = 0.25–0.89). Esotropia increased after 2-wall decompression (*p* = 0.001–0.02), and hypotropia increased after 3-wall decompression (*p* = 0.02). Adduction increased but abduction decreased following 2-wall and 3-wall decompression (*p* < 0.05). Infraduction increased following 3-wall decompression (*p* < 0.001). Additionally, the increase in esotropia was significantly correlated with the increase in adduction and with the decrease in abduction (*r* = 0.37–0.63, *p* < 0.05). There were significant correlations between the diameter of the medial rectus and the increase in esotropia, the increase in adduction and the decrease in abduction postoperatively (*r* = 0.35–0.48, *p* < 0.05).

**Conclusions:**

The changes in ocular deviation and duction were different after 1-wall, 2-wall and 3-wall orbital decompression. The increased contractile force of the rectus may be an important reason for strabismus changes after orbital decompression surgery.

Orbital decompression surgery is a crucial treatment for thyroid eye disease (TED). It is effective for treating proptosis, exposure keratopathy and optic neuropathy. Various surgical techniques have been utilized, including medial, inferior, lateral orbital wall removal with or without intraconal fat removal.

Currently, orbital decompression is more focused on the risks of ocular imbalance after surgery. New-onset diplopia (NOD) and worsening of the pre-existing strabismus are the major problems that have been widely studied. Inferomedial decompression is associated with a relatively high risk of consecutive diplopia and globe displacement (ranging from 9.5 to 73%), whereas balanced decompression (ranging from 10 to 45%) and deep lateral decompression (ranging from 2.6 to 8%) are considered to lessen the imbalanced shifting of extraocular muscles (EOM) and to reduce the likelihood of NOD [1–4]. However, studies on the exact changes in ocular deviation and duction after unilateral orbital decompression are lacking. Studying the relationship between ocular duction and deviation may help to understand the biomechanics of ocular imbalance [5, 6].

This study analysed the changes in uniocular deviation and duction after 1-wall, 2-wall and 3-wall decompression surgery, and assessed the correlations between them.

## Patients and methods

The study was conducted at Eye and ENT Hospital of Fudan University between March 2016 and May 2018. The TED patients who underwent orbital decompression surgery, including 1-wall (deep lateral wall), 2-wall (deep lateral and medial wall) and 3-wall (deep lateral, medial and inferior wall) decompression were enrolled. The indications for orbital decompression included disfiguring proptosis, exposure keratopathy and compressive optic neuropathy.

To evaluate the exact changes in ocular deviation and duction following decompression surgeries, only the patients who underwent unilateral surgery were collected, including the patients who had unilateral proptosis, the patients who had asymmetric proptosis and intended to undergo decompression surgeries at different times, and the patients who had unilateral surgery 6 months previously and needed a decompression surgery for the other eye.

The exclusion criteria included a history of orbital surgery of the studied eyes, low vision with an inability to complete the strabismus examinations, and bilateral orbital decompression performed within a short time. The strabismus and motility examinations were performed within 1 week before the surgery and were repeated several months after the surgery. The following data were obtained: gender, age, disease duration, best-corrected visual acuity (logMAR), clinical activity score (CAS), Hertel exophthalmometry, history of thyroid disease, and iodine 131 and corticosteroid treatment.

The study adhered to the tenets of the Declaration of Helsinki and was approved by the Ethics Committee of Fudan University. Informed consent was obtained from all patients in the study.

### Surgical technique

All decompression surgeries were performed by a single surgeon. Deep lateral wall decompression was performed through an eyelid crease incision. The lacrimal gland fossa and the greater wing of the sphenoid bone were removed using a high-speed drill. The outer region of the zygomatic bone lateral to the inferior orbital fissure (basin region) was additionally removed in some patients. The periorbita was incised to allow orbital fat to herniate into the surrounding spaces. Medial wall decompression was performed through a transcaruncular incision. Medial and posterior ethmoidectomy was performed and the periorbita was incised. For inferior wall decompression, a small region of the posteromedial orbital floor was removed. The ethmoid-maxillary bony strut was preserved. The extent of osteotomy was individualized based on the degree of proptosis. For some patients, the inferolateral intraconal fat was removed with volumes ranging from 0.5 to 1.5 ml.

### Ocular deviation and duction examinations

Strabismus examinations were performed by an experienced orthoptist. Objective deviation was measured with the prism test, and subjective deviation was measured using a synoptophore. Horizontal deviation was recorded as a positive value when the operated eye exhibited esotropia compared with the fellow eye and was recorded as a negative value when exotropia was present. Vertical deviation was recorded as a positive value when the operated eye exhibited hypertropia compared with the fellow eye and was recorded as a negative value when hypotropia was present. Torsional deviation was recorded as a positive value when the operated eye exhibited excyclotropia and was recorded as a negative value when incyclotropia was present.

**Fig. 1:**
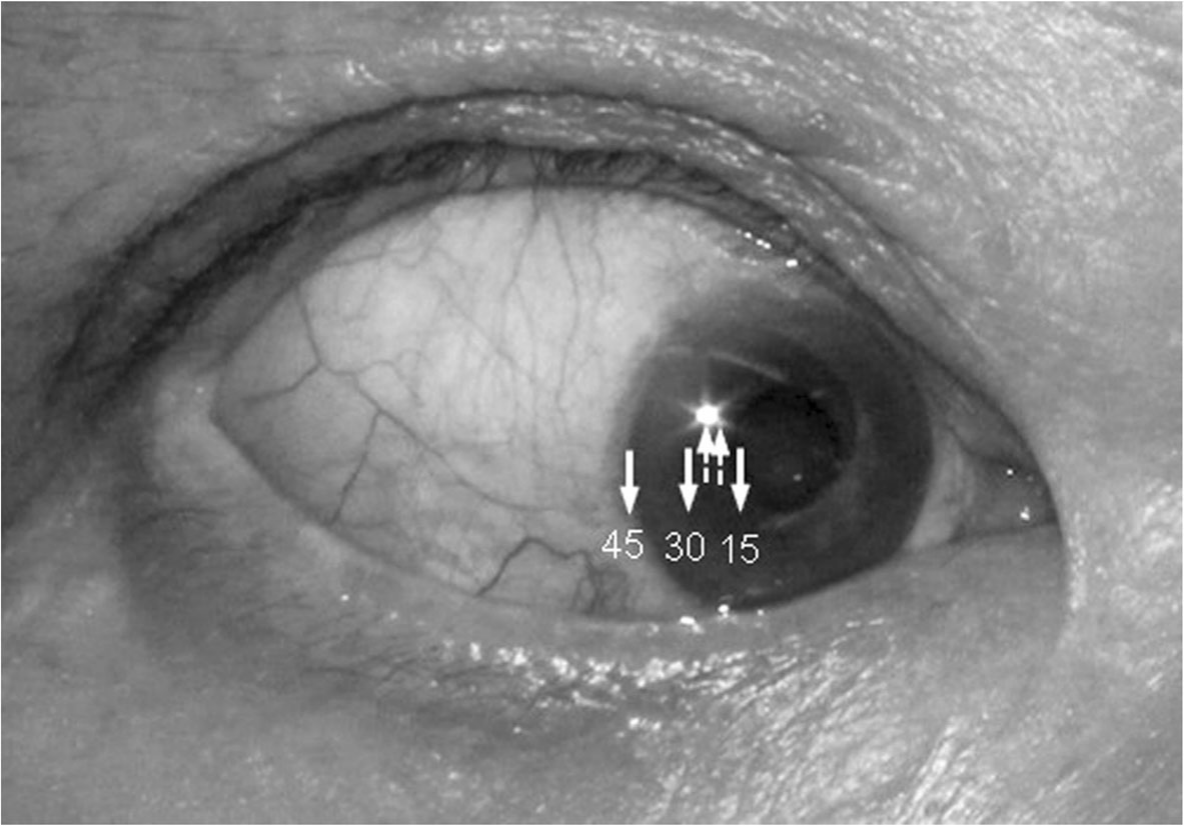
Ocular duction was evaluated using Hirschberg’s method. Light target on the surface of the eye at limbus = 45°, between limbus and pupil = 30°, and at pupil edge = 15° (solid arrow). The space between these designations was divided into thirds, and intermediate duction was estimated to the nearest 5° (dotted arrow). This patient is looking inward at 25°

Ocular duction was evaluated using Hirschberg’s method [7]. The clinician shined a pen light in line with the eye being examined. The patient was asked to look in four cardinal directions as far as possible, and the position of the light was viewed on the surface of the eye: at limbus = 45°, between limbus and pupil = 30°, and at pupil edge = 15°. The clinician estimated 5° intervals by dividing the space into thirds between these designations, and the nearest 5° was recorded (Fig. 1).

### Extraocular muscle evaluations

Computed tomography (CT) scans with 0.75 mm slice thickness were performed within 1 month before surgery. The maximum diameters of the medial rectus (MR) and lateral rectus (LR) were measured from axial CT images. The maximum diameters of the inferior rectus and superior muscle groups, including the superior rectus and levator palpebrae superioris, were measured from coronal CT images.

### Statistical analysis

The mean values are presented with standard deviations. The paired t test was used to compare the preoperative and postoperative measurements. ANOVA was used to compare the preoperative measurements between different groups. Pearson correlation analysis was used to analyse the correlations between ocular deviations, ductions and rectus diameters. The chi-square test was used to compare the decompression extent between the 2-wall and 3-wall groups. All differences with a value of *p* < 0.05 were considered statistically significant. The analyses were performed using SPSS.

## Results

Fifty-four patients (26 men and 28 women) were included in the study. The mean age was 51.7 ± 12.5 years (18–72 years), and the mean CAS was 1.6 ± 1.1 (0–3). Fifteen patients (27.8%) underwent 1-wall decompression surgery, 18 patients (33.3%) underwent 2-wall decompression surgery and 21 patients (38.9%) underwent 3-wall decompression surgery. The mean follow-up duration was 4.2 ± 0.9 months (from 4 to 9 months). The basin region was removed more frequently in the 3-wall decompression group than in the 2-wall decompression group (*p* = 0.003).

The mean proptosis reductions were 2.5 ± 0.5 mm, 3.2 ± 1.1 mm, and 4.4 ± 1.1 mm after 1-wall, 2-wall and 3-wall orbital decompression, respectively. The mean visual acuity improved after 2-wall decompression surgery (logMAR 0.37 ± 0.4 preoperatively and logMAR 0.14 ± 0.14 postoperatively, *p* = 0.04) and 3-wall decompression surgery (logMAR 0.41 ± 0.41 preoperatively and logMAR 0.22 ± 0.25 postoperatively, *p* = 0.001). The mean visual acuity did not significantly change after 1-wall decompression surgery (logMAR 0.04 ± 0.06 preoperatively and logMAR 0.03 ± 0.05 postoperatively, *p* = 0.336).

No significant difference was found in preoperative ocular deviation between the three groups (*p* > 0.05). There was a significant difference in preoperative adduction between the three groups (*p* < 0.05). The patients who underwent 2-wall and 3-wall decompression had more limitations in preoperative abduction than those who underwent 1-wall decompression (*p* < 0.05). The patients who underwent 3-wall decompression had more limitations in preoperative infraduction and supraduction than those who underwent 1-wall decompression (*p* < 0.05).

The changes in objective and subjective strabismus following different orbital decompression are listed in Table 1. Horizontal and vertical deviation showed no significant difference after 1-wall decompression (*p* = 0.25–0.89). Most of the patients who underwent 1-wall decompression had a minor change in horizontal deviation, except for two patients who had enlarged MR preoperatively. Esotropia increased significantly in the patients who underwent 2-wall decompression (*p* = 0.001–0.02), and hypotropia increased in most of the patients who underwent 3-wall decompression (*p* = 0.02). Esotropia had an increasing tendency after 3-wall decompression but the difference did not reach statistical significance. Torsional deviation showed no significant difference postoperatively in any of the groups (*p* = 0.39–0.73).

**Table 1:** Preoperative and postoperative ocular deviation in different orbital decompression surgeries

		1-wall decompression				2-wall decompression				3-wall decompression			
		Pre	Post	Diff	p	Pre	Post	Diff	p	Pre	Post	Diff	p
**Prism test** **(PD)**	**Horizontal deviation**	3.8 (18.1)	9.5 (32.8)	4.1 (14.8)	0.25	6.5 (14.4)	22.5 (26)	16 (14.6)	***0.02***	16.6 (41.3)	26.6 (36.9)	10 (36.3)	0.29
	**Vertical deviation**	−17 (32.9)	−15.2 (33.3)	1.8 (8.8)	0.51	0.9 (8.5)	3.7 (5.8)	2.9 (5.2)	0.17	4.7 (16.6)	−3.5 (10.3)	−8.1 (12.1)	***0.02***
**Synoptophore** **(degree)**	**Horizontal deviation**	7.5 (9.2)	9.7 (16.2)	2.5 (7.6)	0.35	4.9 (8.7)	11.3 (9.8)	6.4 (4.9)	***0.001***	9.9 (12.8)	15.4 (13.8)	5.6 (11.6)	0.07
	**Vertical deviation**	−0.05 (19.5)	0.5 (16.3)	0.5 (11)	0.89	−0.9 (11.3)	−1.4 (8.8)	−0.9 (3.9)	0.6	2.8 (12.1)	−3 (6.2)	−5.8 (10.7)	***0.02***
	**Torsional deviation**	3.1 (6.9)	3.4 (5.9)	0.3 (2.6)	0.73	1.5 (3.1)	2 (4.3)	0.5 (2.1)	0.49	0.9 (4.1)	0.1 (4.1)	−0.8 (3.5)	0.39

The values are the mean (SD). pre = preoperative, post = postoperative, diff = difference, *p* = *p*-value. The significant differences (*p*<0.05) between pre- and postoperative measurements are indicated in bold italic font

**Fig. 2:**
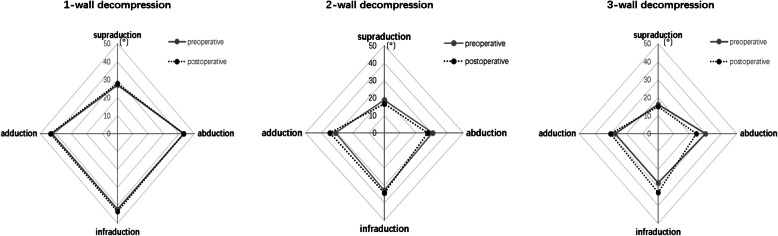
The changes in ocular ductions after different orbital decompression

The changes in ocular ductions after different orbital decompression are shown in Table 2 and Fig. 2. There was no significant change in ocular motility after 1-wall decompression (*p* = 0.083–0.667). Adduction significantly increased after 2-wall and 3-wall decompression (*p* < 0.05), whereas abduction significantly decreased (*p* < 0.05). Infraduction significantly increased after 3-wall decompression (*p* < 0.001).

**Table 2:** Preoperative and postoperative ocular motility in different orbital decompression surgeries

	1-wall decompression				2-wall decompression				3-wall decompression			
	pre	post	diff	p	pre	post	diff	p	pre	post	diff	p
**Supraduction**	27.1 (17.1)	27.9 (17)	0.8 (1.9)	0.083	18.9 (14.3)	16.6 (13.9)	−2.3 (7.4)	0.153	16.2 (12.7)	15 (13.8)	−1.2 (5)	0.286
**Infraduction**	42.1 (14.4)	43.4 (12.7)	1.3 (4)	0.172	32.8 (16.6)	34.4 (14.2)	1.6 (4)	0.08	27.4 (14.8)	32.9 (14.5)	5.5 (5.2)	***< 0.001***
**Adduction**	42.4 (13.6)	43.4 (12.4)	1 (2.7)	0.104	31 (14.9)	34.5 (14.8)	3.5 (5.3)	***0.005***	27.4 (13.4)	30.2 (14.1)	2.9 (5.1)	***0.019***
**Abduction**	43.2 (16.7)	42.9 (17.6)	0.3 (2.6)	0.667	30.7 (12.7)	27.6 (14.2)	−3.1 (6.4)	***0.04***	30 (14.3)	24.3 (15.4)	−5.7 (8.3)	***0.005***

The values are the mean (SD). pre=preoperative, post=postoperative, diff=difference, p=p-value. The significant differences (*p*<0.05) between pre- and postoperative measurements are indicated in bold italic font

**Fig. 3:**
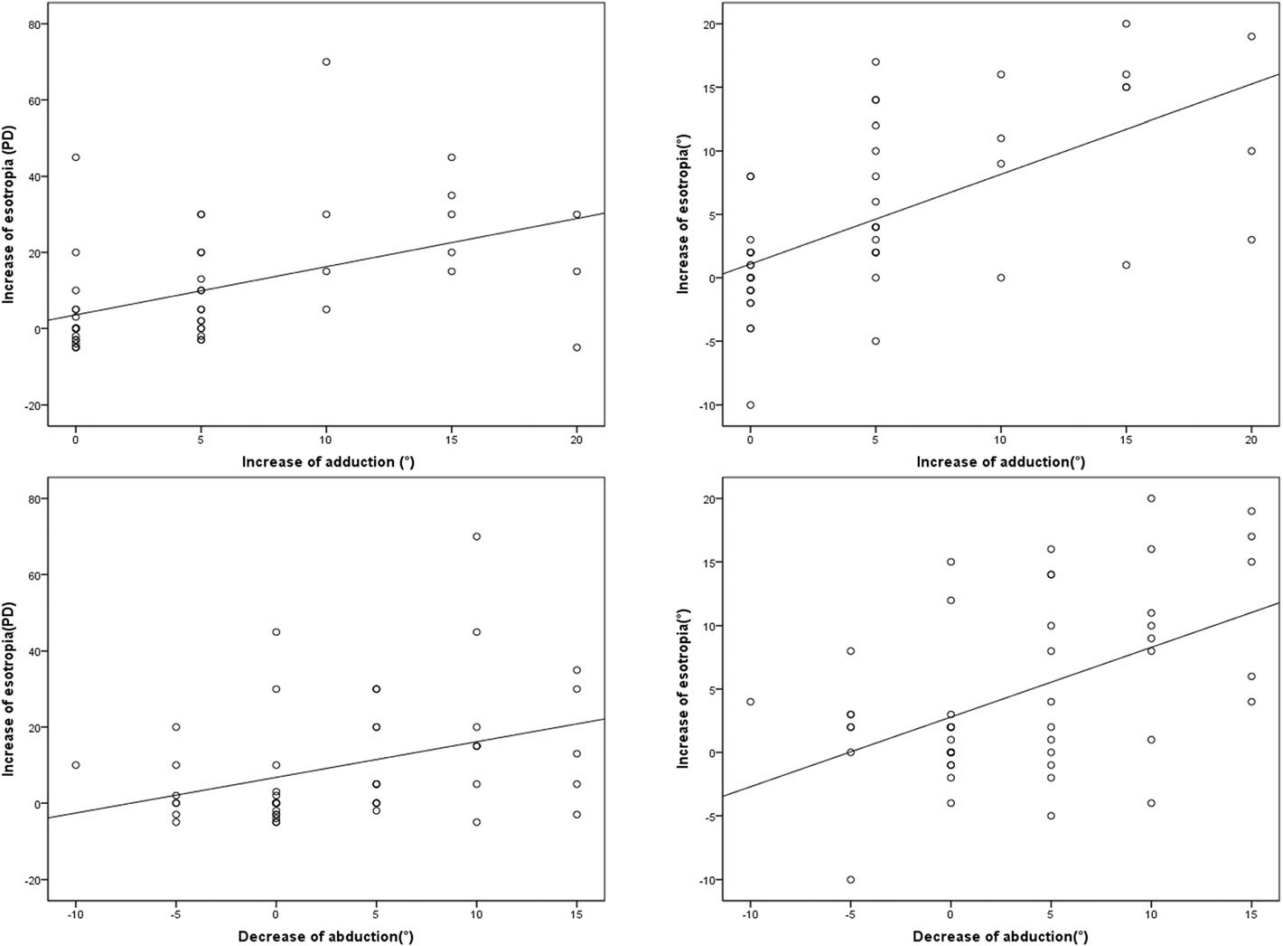
The relationship between the changes in ocular ductions and deviations after orbital decompression. The increase in esotropia had a significant correlation with the increase in adduction and the decrease in abduction

For all the patients who underwent orbital decompression, the increase in postoperative esotropia had a significant correlation with the increase in adduction (prism test: *r* = 0.48, *p* < 0.001, synoptophore: *r* = 0.63, *p* < 0.001) and with the decrease in abduction (prism test: *r* = 0.37, *p* = 0.008, synoptophore: *r* = 0.49, *p* < 0.001) (Fig. 3). Additionally, there were significant correlations between the diameter of MR and the increase in esotropia (prism test: *r* = 0.47, *p* < 0.001, synoptophore: *r* = 0.48, *p* < 0.001), the increase in adduction (*r* = 0.4, *p* = 0.004) and the decrease in abduction (*r* = 0.35, *p* = 0.011). No significant difference was found between the changes in vertical deviations and ocular ductions (*p* > 0.1).

**Fig. 4:**
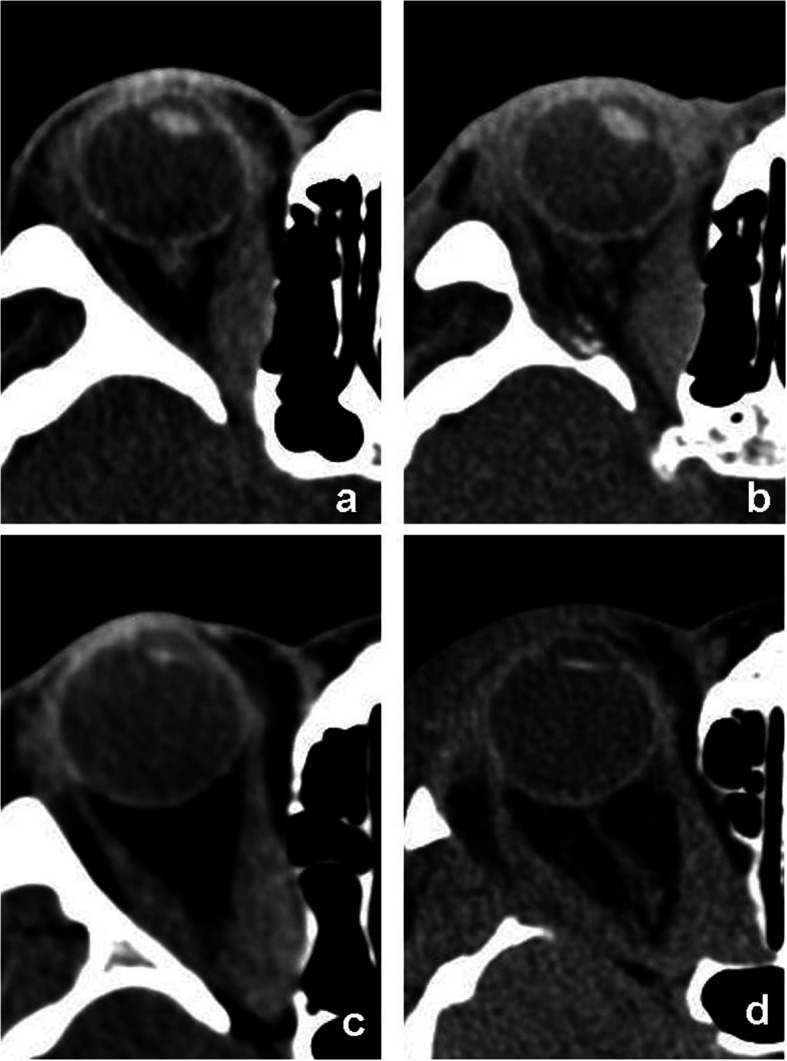
Pre- and postoperative CT images of the patients who underwent orbital decompression. **a-b** A subject who underwent 1-wall decompression had an obvious increase in esotropia, the medial rectus became thicker after surgery. **c-d** A subject who underwent 2-wall decompression had no worsening of strabismus. The position of the medial rectus changed but the thickness did not change much

In some subjects who had an obviously increased esotropia postoperatively, the MR tended to become thicker after surgery, whereas its position exhibited no obvious change (Fig. 4).

## Discussion

Previous reports on strabismus changes following orbital decompression primarily investigated patients who underwent bilateral surgeries. Our study provided an evaluation of the postoperative changes in unilateral ocular deviation and duction, which may aid in the understanding of the biomechanics of ocular imbalance.

Simon et al. reported that the number of patients with esotropia increased after deep lateral decompression [8], and new-onset esotropia was reported to range from 10 to 18 PD [9]. Goldberg et al. suggested that esotropia could worsen after lateral decompression due to LR weakness [10]. However, in another of their studies, the patients exhibited minor exotropia after deep lateral decompression with an average value of 3.7 PD [3]. Additionally, Shani Golan et al. found that the changes in strabismus varied after inferolateral decompression [11]. These discrepancies resulted from different disease severities, preoperative strabismus and surgical techniques. In our study, most of the patients who underwent 1-wall decompression had only a minor change in horizontal deviation except for two patients who had enlarged MR.

New-onset esotropia has been reported to occur after balanced decompression, with the incidence ranging from 4 to 20 PD [9]. In our study, esotropia increased in nearly all patients who underwent 2-wall decompression. Esotropia had an increasing tendency after 3-wall decompression but the difference did not reach statistical significance. The volume of bone removed from the lateral wall was expanded in most patients who underwent 3-wall decompression, including the basin region [10]. We supposed that the expansion of lateral wall decompression could further compensate for the imbalanced shift in orbital tissue and reduce the potential esotropia. Additionally, the preoperative adduction was more restricted in the patients who underwent 3-wall decompression, and the increased stiffness and contractile force of LR could be another reason for the reduction in the postoperative esotropia. A small but significant increase in hypotropia was observed in nearly all patients who underwent 3-wall decompression. Although the bone removed from the orbital floor was limited to the most posteromedial portion adjacent to the ethmoid bone, and the strut was preserved in our study [12].

Although slight differences existed between the results of the synoptophore and prism test due to binocular fusion, accommodation and the kappa angle, the synoptophore test may be better related to the subjective symptom of diplopia than the prism test and could be considered an important component of strabismus evaluation in TED. The change in torsional deviation following orbital decompression has not been discussed previously. Our study showed that torsional deviation did not significantly change after orbital decompression.

The development of oculomotor imbalance following orbital decompression is a multifactorial problem. Michael et al. reported a significant decrease in abduction after 3-wall decompression [13]. Inna et al. reported that adduction and abduction decreased after coronal 3-wall decompression, whereas infraduction decreased after swinging eyelid 3-wall decompression, possibly due to the difference in decompression extent [14]. Rootman et al. reported that abduction worsened after orbital decompression [15]. In our study adduction increased but abduction decreased after 2-wall and 3-wall decompression. The infraduction increased after 3-wall decompression.

It has been proven that the force and elasticity of the extraocular rectus change in TED patients, as well as that the limitation of abduction is not only due to the fibrosis of MR but also to the increased contractile force of MR [16, 17]. In previous studies, the centrifugal displacement of the rectus path was believed to be the main reason for the motility disturbance after decompression [13], however, we found that the MR became thicker without displacement in some patients who had an obvious esotropia postoperatively, which may indicate the increased contractile force of the MR pulling the globe after surgery. That may be why the increase in esotropia correlated with the increase in adduction and the decrease of abduction in the study. Additionally, there were significant differences in preoperative ductions between the different surgical groups. More restriction in preoperative duction indicated greater fibrosis and a greater contractile force of the rectus, and this may be another reason for the different changes in strabismus and motility after decompression surgeries besides decompression extent.

Additionally, we found that a significant correlation existed between MR diameter and the increase in adduction and decrease in abduction. In previous studies, a strong trend toward increased motility restriction with increased muscle diameter was observed in TED patients [18]. MR was more frequently involved than LR in TED, which may be why esotropia increased more frequently after decompression. No significant difference was found between the change in vertical deviation and ocular duction, possibly due to the small sample and the limited extent of inferior wall decompression.

The main limitations of this study were the sample size and the relatively short follow-up, as many patients required bilateral decompression surgeries simultaneously or within a short period of time. The change in ocular deviation during the postoperative recovery period was demonstrated to be typically small [15].

In conclusion, the changes in ocular deviation and duction were different after 1-wall, 2-wall and 3-wall decompression. The increased contractile force of the rectus may be an important reason for the strabismus changes after orbital decompression.

## Data Availability

All data generated or analysed during this study are included in this published article.

## Abbreviations

TED: Thyroid eye disease
NOD: New-onset diplopia
EOM: Extraocular muscles
CAS: Clinical activity score
CT: Computed tomography
MR: Medial rectus
LR: Lateral rectus
